# The Gonadotropin-Inhibitory Hormone: What We Know and What We Still Have to Learn From Fish

**DOI:** 10.3389/fendo.2019.00078

**Published:** 2019-02-19

**Authors:** María P. Di Yorio, José A. Muñoz-Cueto, José A. Paullada-Salmerón, Gustavo M. Somoza, Kazuyoshi Tsutsui, Paula G. Vissio

**Affiliations:** ^1^Departamento de Biodiversidad y Biología Experimental, Facultad de Ciencias Exactas y Naturales, Universidad de Buenos Aires, Buenos Aires, Argentina; ^2^Instituto de Biodiversidad y Biología Experimental y Aplicada (IBBEA), CONICET-Universidad de Buenos Aires, Buenos Aires, Argentina; ^3^Department of Biology, Faculty of Marine and Environmental Sciences, University of Cádiz, Puerto Real, Spain; ^4^Marine Research Institute (INMAR), Marine Campus of International Excellence (CEIMAR) and Agrifood Campus of International Excellence (ceiA3), Puerto Real, Spain; ^5^Instituto Tecnológico de Chascomús (CONICET-UNSAM), Chascomús, Argentina; ^6^Department of Biology and Center for Medical Life Science, Waseda University, Tokyo, Japan

**Keywords:** GnIH, fish, brain, ontogeny, GnRH, growth, reproduction

## Abstract

Gonadotropin-inhibitory hormone, GnIH, is named because of its function in birds and mammals; however, in other vertebrates this function is not yet clearly established. More than half of the vertebrate species are teleosts. This group is characterized by the 3R whole genome duplication, a fact that could have been responsible for the great phenotypic complexity and great variability in reproductive strategies and sexual behavior. In this context, we revise GnIH cell bodies and fibers distribution in adult brains of teleosts, discuss its relationship with GnRH variants and summarize the few reports available about the ontogeny of the GnIH system. Considering all the information presented in this review, we propose that in teleosts, GnIH could have other functions beyond reproduction or act as an integrative signal in the reproductive process. However, further studies are required in order to clarify the role of GnIH in this group including its involvement in development, a key stage that strongly impacts on adult life.

## Introduction

In 2000, Tsutsui's group isolated, for the first time, a novel hypothalamic neuropeptide from the brain of the Japanese quail, *Coturnix japonica*, which inhibited luteinizing hormone (LH) release from the anterior pituitary and named it gonadotropin-inhibitory hormone (GnIH) (1). This finding had the novelty that, at that moment, it was known that gonadotropin secretion was mainly under the stimulatory effect of gonadotropin-releasing hormone (GnRH), but an inhibitory neuropeptide of gonadotropin secretion had been not discovered. The discovery of GnIH opened a new research field in reproductive neuroendocrinology from a novel standpoint. Since then, GnIH orthologs were described in protochordates (2) and many vertebrate taxa including agnathans, teleosts, amphibians, reptiles, birds, and mammals [for review see (3, 4)]. In some of these groups, it is clear that GnIH is involved in the regulation of reproduction, inhibition of pituitary gonadotropins, and sexual behavior [for reviews see (5–9)]; however, up to this moment this is far to be a common feature. After Tsutsui's first finding (1), Satake et al. (10) characterized in quail a cDNA encoding GnIH and two GnIH-related peptides. Later, in most vertebrate species, these peptides were deduced from the cDNA sequences of their precursors, but the GnIH peptide was isolated and identified only in a few species: quail (1), starlings (11), zebra finches (12), chicken (13), rats (14), Siberian hamsters (15), bullfrog (16), turtles (17), primates (18), humans (19), and goldfish (20). All these GnIH orthologs have an Arg-Phe-amide as a C-terminal sequence and thus, they are part of the RF-amide family. Particularly, GnIH and its related peptides, either putative or identified, possess a common LPXRFamide or MPXRFamide (X: L or Q) C-terminus motif [for reviews see (5–9)].

It is known that more than half of the vertebrate species are teleosts, and teleost-3R genome duplication could have been responsible for the great phenotypic complexity observed in this group of vertebrates (21). They occupy all aquatic environments and present a tremendous variability of reproductive strategies and sexual behavior (22). In this context, fish GnIH and their related peptides could have undertaken new and maybe unexpected functions.

**Figure 1:**
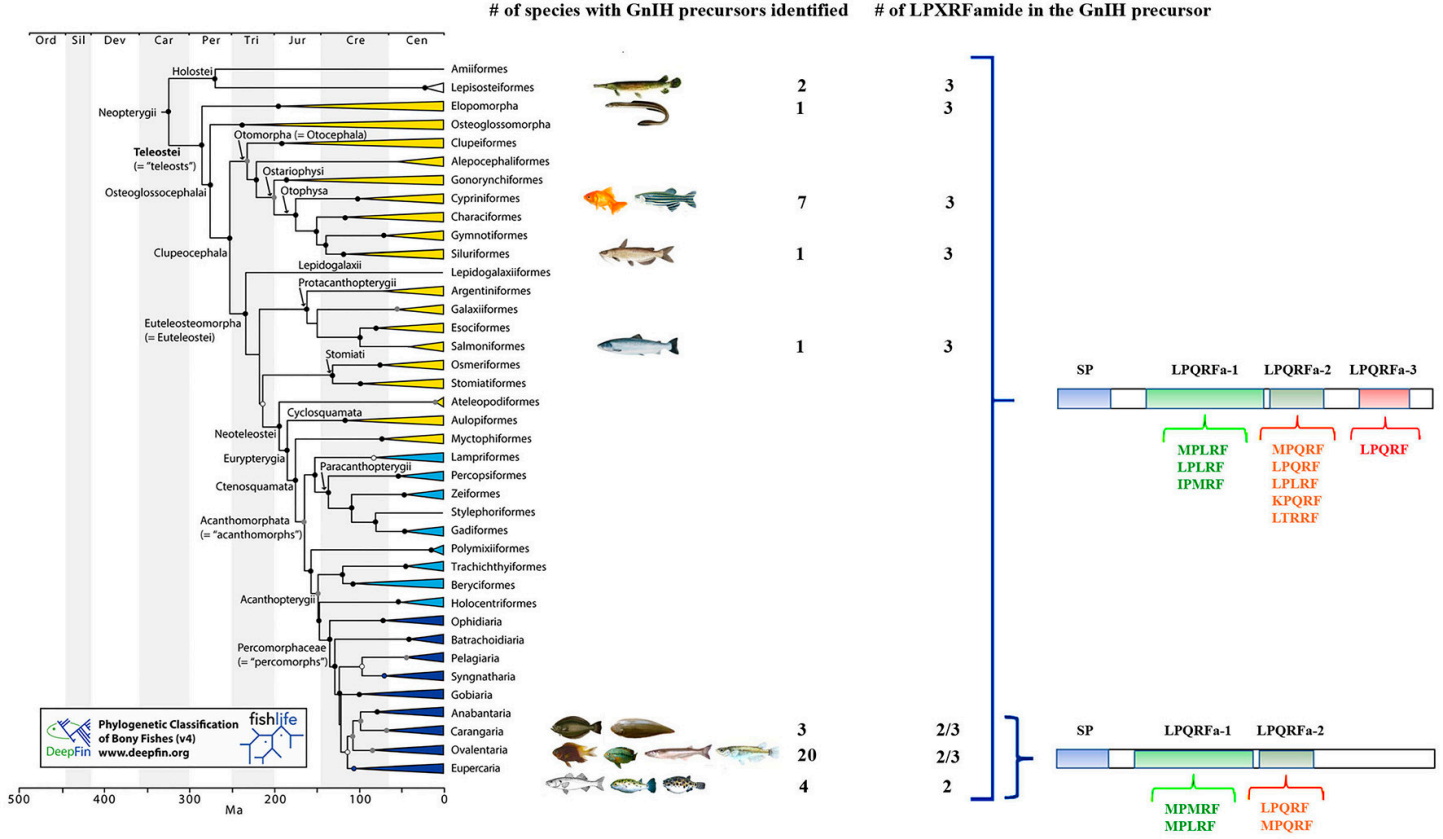
Phylogenetic tree of fish showing the different orders in which GnIH precursor genes have been identified and the number of LPXRFamide peptides present in these GnIH precursors. The amino acid motifs present in the C-terminal region of these LPXRFamide peptides are also presented. SP, signal peptide. The accession numbers of the identified and possible GnIH precursor sequences obtained from US National Center for Biotechnology Information database are: **Lepisosteiformes**: *Atractosteus tropicus* (Álvarez-González, C.A., personal communication; transcriptome sequencing: PRJNA395289), *Lepisosteus oculatus* (XP_015213317.1); **Elopomorpha**: *Anguila japonica* (XP_013998456.1); **Cypriniformes:** *Carassius auratus* (BAC06473.1), *Catla catla* (AUO16369.1), *Cyprinus carpio* (AML83913.1), *Danio rerio* (NP_001076418.1), *Pygocentrus natteri* (XP_017549097.1), *Sinocyclocheilus grahami* (XP_016150344.1), *Sinocyclocheilus rhinocerous* (XP_016370559.1); **Siluriformes:** *Ictalurus punctatus* (XP_017336524.1); **Salmoniformes:** *Salmo salar* (XP_013998456.1); **Carangaria:** *Cynoglossus semilaevis* (AMB48604.1), *Paralichthys orbignyanus* (Mechaly A.S., personal communication), *Solea senegalensis* (24); **Ovalentaria:** *Austrofundulus limnaeus* (XP_013866639.1), *C. dimerus* (25), *Cyprinodon variegates* (XP_015229614.1), *Fundulus heteroclitus* (XP_012729657.1), *Iconisemion striatum* (SBP35361.1), *Kryptolebias marmoratus* (XP_017278134.1), *Neolamprologus brichardi* (XM_006788075.1), *Nothobranchius furzeri* (XP_015811406.1), *Nothobranchius kuhntae* (SBQ91527.1), *Nothobranchius pienaari* (SBR89569.1), *Odontesthes bonariensis* (Somoza G.M., personal communication), *Oreochromis niloticus* (NP_001298256.1), *Oryzias latipes* (XP_004073896.1), *Poecilia formosa* (XP_007562706.1), *Poecilia latipinna* (XP_014884496.1), *Poecilia mexicana* (XP_014852162.1), *Poecilia reticulata* (XP_008419875.1), *Pundamilia nyererei* (XP_013765199.1), *Stegastes partitus* (XP_008290012.1), *Xiphophorus maculatus* (XP_005802819.1); **Eupercaria:** *Dicentrarchus labrax* (CEK03537.1), *Takifugu rubripes* (NP_001092115.1), *Tetraodon nigroviridis* (BAF34880.1), *Thalassomabi fasciatum* (ANV28067.1). The phylogenetic tree was taken from Figure 1 of Betancur et al. (23).

Early-branching lineages of ray-finned fishes (such as gars) and teleosts already present a GnIH precursor suggesting that this peptide emerged before to the teleost whole genome duplication. Early-branching lineages of teleosts, such as anguiliforms and otophysans (Cypriniformes, Characiformes, and Siluriformes), and salmoniforms, ovalentarians (some Cichliformes, Cyprinodontiformes, Atheriniformes, and Beloniformes) (23) and some Pleuronectiformes (24), exhibit a GnIH precursor encoding three peptides. However, in late-branching evolved species belonging to ovalentarians (some Cichliformes, Perciformes), Pleuronectiformes and Tetraodontiformes, two peptides were described (3) (Figure 1). Then, it seems that species belonging to early branching teleost lineages contain GnIH and 2 related peptides, whereas those species belonging to late branching lineages possessed GnIH and 1 or 2 related peptides, suggesting that one was lost in the course of teleost evolution. The meaning of this fact is still unknown, but it represents an interesting matter of study in neuropeptide evolution (3, 25).

## The GnIH System in Fish

### Neuroanatomical Distribution of GnIH Cell Bodies and Fibers

**Figure 2:**
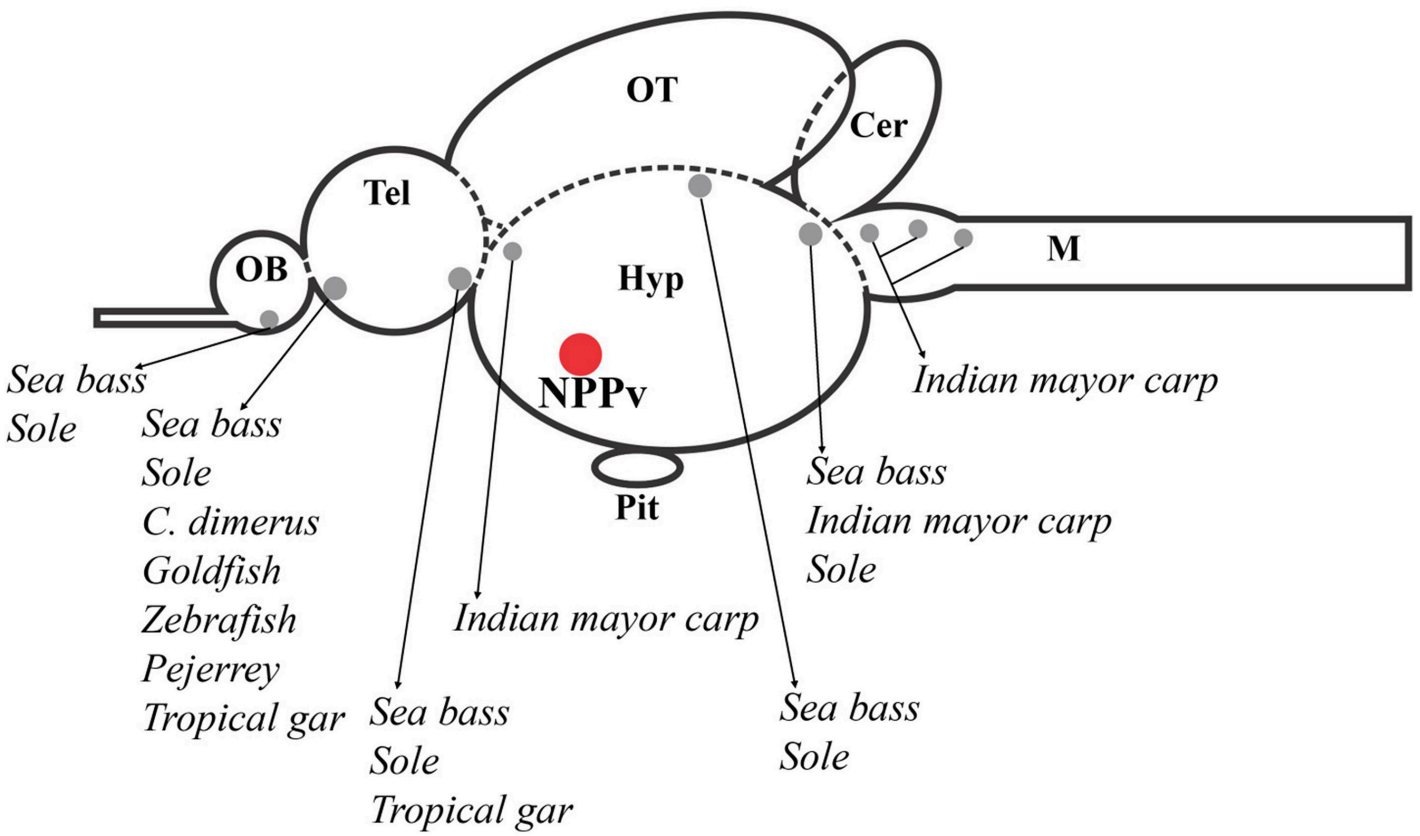
Schematic representation of a sagittal section of the fish brain showing GnIH cell populations described in several brain areas of different fish species, which are represented by gray circles. The red circle indicates the presence of GnIH cells in the nucleus *posterioris periventricularis* (NPPv) that has been reported in all fish species studied until now. OB, olfactory bulb; Tel, telencephalon; Hyp, hypothalamus; OT, optic tectum; Pit, pituitary; Cer, cerebellum; M, medulla.

In order to gain understanding about the GnIH system in fishes, several studies have investigated the precise localization of GnIH-producing cells in the brain and peripheral organs of teleosts by using PCR, *in situ* hybridization and immunohistochemical techniques [for review see (3)]. Although these studies have reported important consistencies in the brain GnIH innervation pattern, the localization of GnIH cell bodies showed considerable dissimilarities in many of the analyzed species. For instance, in sockeye salmon, *Oncorhynchus nerka*, (26) and tilapia, *Oreochromis niloticus* (27), immunohistochemistry revealed the presence of GnIH-immunoreactive (GnIH-ir) cells only in the diencephalic posterior periventricular nucleus (NPPv), whereas studies developed in other species reported the presence of GnIH-ir cell populations also in other brain regions (3). In this sense, increasing evidence obtained in the last years also suggest that GnIH neurons in teleosts are not only restricted to the caudal preoptic area/hypothalamus (24, 25, 28–31), as it occurs in birds and mammals (32) (Figure 2).

Analyzing the cell clusters reported in fish species from the forebrain to the hindbrain, the most rostral GnIH-cell population described is the one present in the olfactory bulb or terminal nerve ganglion cells (TNgc)/the nucleus *olfacto retinalis* (NOR) of goldfish, *Carassius auratus* (20); developing Indian major carp, *Labeo rohita* (33); sea bass, *Dicentrarchus labrax* (29); cichlid fish, *Cichlasoma dimerus* (25); zebrafish, *Danio rerio* (30); sole, *Solea senegalesis* (24), and pejerrey, *Odontesthes bonariensis* (31). This immunostaining was consistent with *gnih* expression detected in these areas of sea bass brain by RT-PCR. Moreover, these results were confirmed by sensitive laser-capture microdissection followed by quantitative real-time PCR (29). Similar analysis and/or *in situ* hybridization studies in this brain region appear necessary to confirm the presence of GnIH in these cells in other teleost species. More recently, immunohistochemical studies also showed that GnIH neurons are located in this transitional region between the olfactory bulbs and telencephalic hemispheres of pejerrey, *Odontesthes bonariensis*, an atheriniform species (31), and the tropical gar, *Atractosteus tropicus*, an ancient lepisosteiform fish (28). More caudally, in the ventral telencephalic area, another GnIH-cell population was described, for the first time, in sea bass (29). Similarly, Aliaga-Guerrero et al. (24) reported the presence of GnIH-ir neurons in the central and lateral subdivision of the ventral telencephalon using specific antibodies developed against sole GnIH.

In the diencephalon, GnIH-ir neurons were detected in the suprachiasmatic nucleus in the tropical gar (28); while, in the India major carp, GnIH-cell masses were observed in the magnocellular preoptic nucleus (NOPm) (33). Among the diencephalic GnIH cell masses identified in different species, the one present in the posterior periventricular nucleus (NPPv) of the caudal preoptic area is the most conserved in all fish species studied so far, including goldfish (20), sockeye salmon (26), Indian major carp (33), orange-spotted grouper, *Epinephelus coioides* (34), sea bass (29), tilapia (27), *Cichlasoma dimerus* (25), pejerrey (31), zebrafish (35), sole (24), and tropical gar (28). Moreover, in agnathans, the most ancient lineage of vertebrates, a lamprey *gnih* precursor mRNA was only expressed in the rostral and caudal regions of the bed nucleus of the tract of the postoptic commissure (nTPOC) in the hypothalamus (36). The presence of other, but more posterior, hypothalamic GnIH-ir cell population was also seen in tropical gar, within the tuberal hypothalamus (28). In addition, GnIH-ir cells were also present in the dorsal mesencephalic *tegmentum*, as well as the rostral rhombencephalon of Indian major carp, sea bass and sole (24, 29, 33). The analysis of *gnih* expression in the mesencephalic *tegmentum* confirmed these neurons as genuine GnIH-expressing cells by using sensitive laser-capture microdissection followed by quantitative real-time PCR in sea bass (29).

The profuse innervation of GnIH cells in the brain is a common feature of all birds and mammals studied so far, as well as in fishes (20, 24–29, 31, 33). This pattern of the distribution of GnIH projections strongly suggests that GnIH acts in many brain sites and then its function can be not only related to reproduction.

### GnIH Fiber Projections to the Pituitary Gland

In fish, GnIH-ir fibers were found running along the ventral hypothalamus, reaching the infundibulum to project into the pituitary [for review see (3)]. It is important to highlight to the readers who are not specialized in fish neuroendocrinology, that nerve terminals of hypophysiotropic neuroendocrine cells from fish can establish direct contacts (like a “synaptic terminal”) or end close to pituitary cells to release their neurohormones (37), and they do not exhibit neither a median eminence nor the portal vasculature reported in tetrapods (38). These fibers were found in the proximal *pars distalis* (PPD) of goldfish (20), sockeye salmon (26), sea bass (29), tilapia (27), zebrafish (35), pejerrey (31), and sole (24) and, more recently, they were also found in the neurohypophysis of the tropical gar (28). The presence of GnIH-ir fibers in the PPD reinforces the role of this neuropeptide in the regulation of pituitary hormone secretion also in fish. Furthermore, GnIH-ir fibers were observed in close proximity to FSH, LH, and GH cells in the pituitary of sea bass (29), and FSH, LH, POMC, and α-MSH cells in the pituitary of tilapia (27). Nevertheless, GnIH-pituitary innervation has not been demonstrated in adult specimens of other fish species, such as the Indian mayor carp and *C. dimerus* (25, 33). However, we cannot discard that a sexual-stage-dependent plasticity in the GnIH-pituitary innervation, or a neurovascular supply to the pituitary, exists as it was reported in zebrafish (39).

### GnIH and Photoperiodic Control of Reproduction

Much has been written about the role of the GnIH system in transducing and/or mediating photoperiodic effects on reproduction through its interactions with the pineal organ and retina in vertebrates (40–43). The pineal organ of fish is a light-sensitive structure responsible for the nocturnal production of melatonin, playing a central role in the transduction of daily and seasonal information (44). To date in fish, only a few studies have investigated the links between GnIH and melatonin (45–49). In cinnamon clownfish, *Amphipirion melanopus*, it was shown that GnIH and the melatonin receptor MT1 co-localized in diencephalic cells (46). Moreover, it has been reported the existence of day-night differences in the expression of *gnih* in sea bass, suggesting a role of melatonin in the modulation of the GnIH system in this species (50). Accordingly, GnIH fibers were localized in the pineal organ of sea bass (29), sole (24), pejerrey (31), and tropical gar (28), suggesting the existence of bidirectional connections between the pineal organ and GnIH cells. Besides GnIH-ir fibers were found in the vascular sac of sea bass (29), sole (24) and *C. dimerus* (51), an organ that represents a sensor of daily and seasonal changes in day length and has been involved in the photoperiodic control of reproduction and other rhythmic processes in some teleost species (52). The interactions between GnIH and both sensor systems (pineal organ and vascular sac) could imply a role of GnIH in the relay between environment and seasonal reproduction in this group of vertebrates. Other studies observed that *gnih* was expressed in the retina of different fish species, such as sea bass (29), zebrafish (30), sole (24), and *C. dimerus* (51). However, only one report relating retinal GnIH with the reproductive cycle has been published so far, showing a decrease in retinal *gnih* expression in late-vitellogenic zebrafish females (30). The fact that GnIH is expressed in the retina could indicate its modulatory role in this photosensory organ, but further studies appear necessary to clarify the physiological significance of this GnIH action.

### GnIH Receptors

The study of distribution of GnIH receptors (GnIH-R) has provided relevant information to recognize the neural targets of GnIH cells, helping to identify new putative roles of this neuropeptide in the brain and peripheral organs. Unfortunately, the precise identification of GnIH-R containing cells is still scarce and only a few studies have used molecular tools and antibodies to address its detailed localization in the fish brain. Both GnIH-ir fibers and GnIH-R were widely distributed in the tilapia brain but they were particularly evident in cells bodies of the preoptic area, hypothalamus, optic tectum, semicircular torus, and caudal midbrain *tegmentum*. They also coexist in the olfactory bulbs, ventral/dorsal telencephalon and in the rhombencephalon (27). In addition, GnIH-R immunoreactivity was found in LH, ACTH, and α-MSH cells of tilapia pituitary (27). Moreover, three different GnIH-R subtypes have been identified in goldfish (53) and zebrafish (54), but their presence in other fish is still not reported. In goldfish, three subtypes of GnIH-Rs were localized in neuroendocrine regions as the preoptic area and the NPPv, the *preoptic nucleus* (NPO), and the lateral *tuberal nucleus* (NLT), whereas only two GnIH-R subtypes (GnIH-R1 and GnIH-R2) were observed in the *pars intermedia* of the pituitary gland. Surprisingly, no signals of GnIH-R were observed in the proximal and rostral *pars distalis* of the goldfish pituitary (54). On the other hand, the presence of *gnih-r* transcripts was also revealed in the brain and pituitary of zebrafish (53), grass puffer, *Takifugu niphobles* (45), and tongue sole, *Cynoglossus semilaevis* (55), by using RT-PCR. In addition, RT-PCR and *in situ* hybridization studies have reported the expression of *gnih* and *gnih-r* in some peripheral fish organs, including the gonads (24, 25, 29, 30, 34, 45, 46, 53, 54, 56–58), which could indicate an autocrine/paracrine role of GnIH in gonadal function.

## GnIH and GnRH Relationships

It is well-established that in vertebrates, multiple GnRH variants are expressed by different neurons in the brain of a single species. These variants are currently classified into three different types, according to their amino-acid sequence, neuroanatomical localization, embryological origin, and synteny: GnRH1, GnRH2, and GnRH3 [for review see (59)]. In the case of teleost fish, GnRH1, the most variable GnRH type according to its amino-acid sequence, is expressed in neurons originated from the olfactory placode during embryogenesis (59–62) and plays the classical hypophysiotropic function in most species. GnRH2 is mainly produced by midbrain *tegmental* neurons and it has been proposed that plays a key role in reproductive behavior [for review see (63)]. Finally, GnRH3 is expressed in ventral forebrain neurons, from the olfactory bulbs to the hypothalamus, and seems to act as a neuromodulator of olfactory and visual information related to reproduction (64, 65). This variant also plays hypophysiotropic functions especially in those teleost species expressing two GnRH variants: GnRH2 and GnRH3 as most of Cypriniformes and Salmoniformes (38, 66).

In birds and mammals, GnIH regulates gonadotrophs' function either directly or indirectly via GnRH neurons (11, 67–69). Considering that GnRH is the key neuropeptide in the control of gonadotropin synthesis and secretion, it could be the candidate through which GnIH acts in fish. In this conceptual frame, several studies have analyzed the relationship between both systems, although most of the mare focused on physiological approaches. Even though it could be considered that morphological associations may allow us to infer physiological interactions, there is scarce information on the relationship between GnIH and GnRH neurons in fishes.

### GnIH-GnRH1 Neuroanatomical Interactions

As it was previously mentioned, GnRH1 is the main hypophisiotropic variant in most fishes, but neither in tilapia (27) nor in *C. dimerus* (70), axo-somatic or fiber-fiber contacts were observed between GnRH1 and GnIH neurons; although, in *C. dimerus* GnIH axons were detected in close proximity to GnRH1 fibers. Additionally, in sea bass (71) and zebrafish (35), GnIH terminals contacted GnRH1 cells or GnRH3 in the preoptic area, respectively. Taking into consideration these results, it is possible that either there are interspecific differences in this interaction, or it shows plasticity depending on the sexual stage, as it was suggested in sea bass (71) and *C. dimerus* (70). Another possibility is that GnIH can modulate other neurons, as those producing kisspeptin, dopamine or neuropeptide Y, to control gonadotropin secretion. For instance, in zebrafish, GnIH-immunoreactive fibers were observed interacting with kisspeptin receptor-1a-expressing neurons in the preoptic area (35), and a GnIH innervation on Kiss2 cells of the nucleus of the lateral recess has been reported in sea bass (71), although in tilapia, GnIH cells do not seem to be connected with either GnRH1, GnRH3 or kisspeptin neurons (27).

### GnIH-GnRH2 Neuroanatomical Interactions

In Indian major carp (33), sea bass (29), and sole (24) a cluster of GnIH somas were localized in the midbrain; however, this is not a common feature of all analyzed species. Only one study reported the GnRH2 and GnIH relationship and demonstrated fiber-to-fiber contacts in the nucleus *lateralis tuberis* and the midbrain *tegmentum*, suggesting a possible regulation between them (70). This GnIH-GnRH2 association could represent the morphological substrate of a network mediating the transduction of environmental information to the reproductive axis. This is further supported by the interactions among GnRH2, GnIH and melatonin reported in several fish species (24, 29, 46–48, 72, 73).

### GnIH-GnRH3 Neuroanatomical Interactions

**Figure 3:**
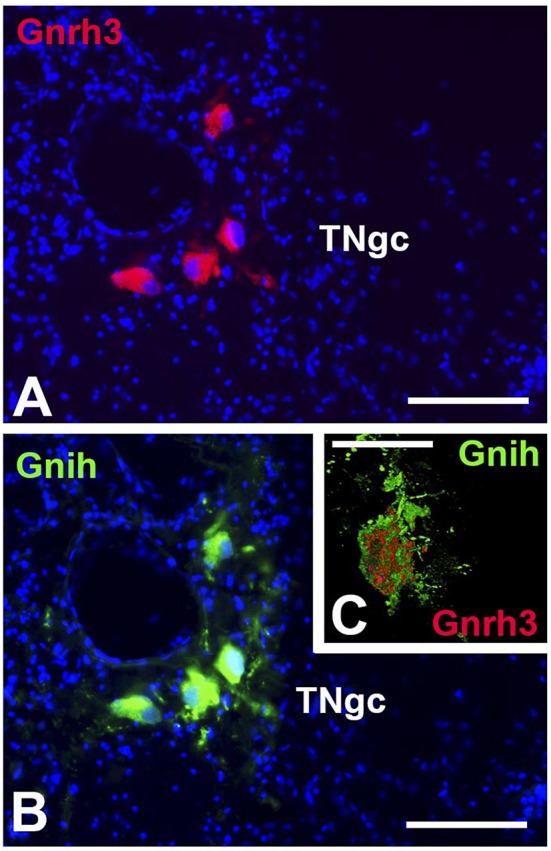
Coexpression of GnIH and GnRH3 in the terminal nerve area of sea bass. GnRH3 positive immunolabeling (red, **A**) and GnIH positive immunolabeling (green, **B**) in the same cell bodies of the terminal nerve region, presenting evidence of co-expression of these two neurohormones. Images **(A,B)** were captured on a conventional fluorescence photomicroscope. **(C)** High resolution confocal image presenting evidence for the co-localization of GnIH (green) and GnRH3 (red) in the same cell bodies, but packaged in separate neurosecretory vesicles. In A and B, the nuclei of cells are stained with DAPI (blue). TNgc: terminal nerve ganglion cells. Scale bars = 100 μm in **(A,B)**, and 50 μm in **(C)**.

**Figure 4:**
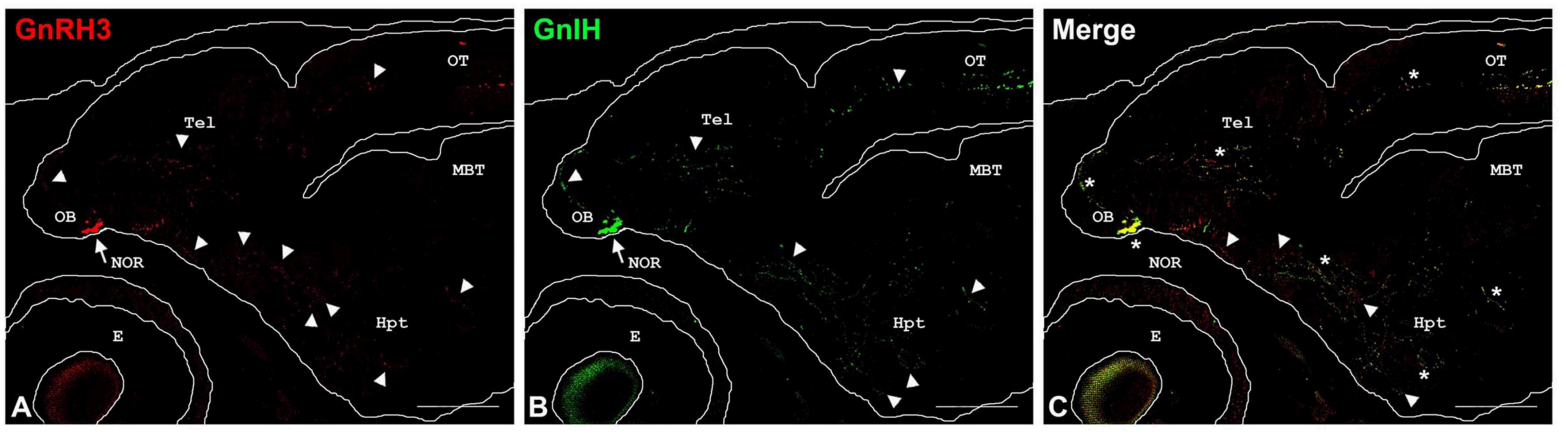
Double-labeling immunofluorescence in parasagittal brain sections of 27 days post-hatching *C. dimerus* larvae using GnIH and GnRH3-GAP antisera. Microphotographs present GnRH3-GAP-immunoreactive (GnRH3) neurons (red, **A**) and GnIH-immunoreactive (GnIH) neurons (green, **B**). In **(C)**, a merge image of **(A,B)** is presented. Immunoreactive cell somata are indicated by arrows and some representative fibers appear marked by arrowheads. Co-localization is indicated by asterisks. Scale bar: 200 μm. OB, olfactory bulb; NOR, *nucleus olfacto retinalis*; Tel, telencephalon; Hyp, hypothalamus; OT, optic tectum; MBT, midbrain tegmentum; E, eye.

Finally, GnRH3 and GnIH neurons were observed in the TNgc of most fish species [for review see, (20, 24, 25, 29, 30, 53, 62)]. The co-localization of both peptides in the same neurons was observed in *C. dimerus* since early developmental stages (51) and in adults of this species (70), pejerrey (31), and sea bass (Figures 3A,B). Moreover, a deeper study on co-localization showed, for the first time, that GnIH and GnRH3 peptides are localized in different neurosecretory vesicles (Figure 3C), suggesting that both peptides can be independently regulated and secreted. It is also interesting to notice that in most of the brain regions analyzed in *C. dimerus*, some fibers co-expressed GnRH3 and GnIH; whereas some other fibers only expressed GnRH3 or GnIH, suggesting that these fibers correspond to neurons located in other brain areas as the NPPv (for GnIH) or from the OB, ventral TEL and POA (for GnRH3) (Figure 4). However, no contacts between GnRH3 fibers and GnIH neurons were observed (70).

### Physiological Interactions Between GnIH and GnRHs

The physiological action of GnIH on GnRH synthesis and its modulating effects over GnRH-stimulatory action on the synthesis and release of gonadotropins or GH are summarized in Table 1. From these data, GnIH can prevent the GnRH-stimulatory action on the synthesis and/or release of FSH, LH, and GH. For example, Moussavi et al. (74, 75) showed that when GnRH2 or GnRH3 are co-administrated with goldfish GnIH-III (gGnIH-III), the stimulatory action of GnRH2/GnRH3 on LH secretion was attenuated by goldfish GnIH-III, together with *lh*β *or fsh*β synthesis, especially during mid and late recrudescence (74); while gGnIH-III prevented GnRH2/GnRH3 stimulation of *gh* transcript levels and GH secretion (75). Moreover, gGnIH-III differentially affected GnRH2 and GnRH3 actions depending on the sexual stage. Similar effects of GnIH peptides were demonstrated in *Amphiprion melanopus* (46) and in *Astyanax altiparanae* (78). This preventive-GnRH-stimulatory effect has been proposed to depend on the action of estradiol or neuroestrogen levels; the abundance of GnIH-R, GnRH and estrogen receptors, and the inhibition of cAMP pathways or the hyperpolarization of gonadotropes by activating K^+^, both exerted by the activation of GnIH-R. Other possibility is that GnIH-R and GnRH-R could form heterodimers modifying the action of their ligands on gonadotrophs [for review see (79)]. Although these statements have been mostly established in birds and mammals (80, 81), there is evidence that a similar mechanism could be operating in teleosts (34, 55, 82, 83).

**Table 1:** Physiological actions of GnIH on GnRH in fish.

***In vivo* or *in vitro* experiment**	**Species**	**Effect**	**Animals physiological status**	**Authors**
Two ip. injections of gGnIH-III (0 or 2 μg/fish) plus GnRH3 or cGnRH2 (0 or 4 μg/fish).	*Carassius auratus*	Seasonal dependent action. gGnIH-III often prevented GnRH3/GnRH2-stimulated LH secretion, or *lhβ* and *fshβ* synthesis.	Mixed sex in early, mid, or late recrudescence.	Moussavi et al. (74)
Ip. injection of gGnIH-II or gGnIH-III (0 or 100 ng/g of BW).	*Carassius auratus*	Both gGnIH-II and -III inhibited only hypothalamic *gnrh3* levels	Females in late vitellogenic stage.	Qi et al. (54)
Primary pituitary cell culture in the presence of gGnIH-II or -III (0 or 100 nM) in combination with GnRH-A (0 or 100 nM).		gGnIH-III prevented GnRH-A-stimulated *fshβ* synthesis		
Two ip. injections of gGnIH-III (0 or 2 μg/fish) plus GnRH3 or GnRH2 (0 or 4 μg/fish).	*Carassius auratus*	Seasonal dependent action. gGnIH-III often prevented GnRH3/GnRH2-stimulated GH secretion, or on *gh* synthesis.	Mixed sex in early, mid, or late recrudescence.	Moussavi et al. (75)
Primary pituitary cell static culture in the presence of gGnIH-III (0–100.nM) plus GnRH3 (0 or 10 nM), or pituitary cell column perfusion experiments exposed togGnIH-III (0 or 10 nM) and GnRH2 or GnRH3 (0 or 100.nM).		Seasonal dependent action. gGnIH-III often attenuated GnRH3/GnRH2-stimulated GH secretion, or on *gh* synthesis.		
Two ip. injection of grGnIH-I, grGnIH-II, or grGnIH-III (0 or 100 ng/g of BW).	*Epinephelus coioides*	Hypothalamic *gnrh1* levels were reduced by all grGnIH peptides, and only grGnIH-III increased *gnrh3* synthesis.	Females.	Wang et al. (34)
Ip. injections of gGnIH-III (0 or 0.1 μg/g of BM) in combination with GnRH1 (0 or 0.1 μg/g of BM).	*Amphiprion melanopus*	gGnIH-III decreased *gnrh1* brain expression levels and its secretion 24 h post-injection. gGnIH-III attenuated GnRH1 stimulatory effect on *gnrh1, gthα, fshβ, and lhβ* levels, and on GnRH1, FSH, and LH secretion.	Immature fish, males, and females.	Choi et al. (46)
Icv. injection of sbGnIH-I, or sbGnIH-II (0,1, 2 or 4 μg/fish).	*Dicentrarchus labrax*	sbGnIH-1 decreased *gnrh1* brain expression levels at all doses tested. sbGnIH-2 decreased brain *gnrh2*and pituitary *gnrhr-II-1a* synthesis at all doses tested.	Males at the beginning of the reproductive period.	Paullada-Salmeron et al. (76)
Im. injection, one time per month for 5 months, of sbGnIH-I or sbGnIH-II (0 or 1 μg/g of BW).	*Dicentrarchus labrax*	Only sbGnIH-2 administration increased brain *gnrh2*.	Adult males treated during gametogenesis.	Paullada-Salmeron et al. (77)
Brain slides (200-300 μm thickness) incubated with 0,0.1,0.5,1,5 nM of zGnIH-III	*Danio rerio*	zGnIH-III reduced *gnrh3* expression levels at all concentration tested, and *gnrh2* was increased by zGnIH-III at 0.1 nM.	Adult males.	Spicer et al. (35)
Im. injection of ssGnIH-II, or ssGnIH-III (0,0.1, 1 μg/g of BW).	*Solea senegalensis*	ssGnIH-3 reduced *gnrh3* expression levels 4 h post-injection of 1 μg/g of BW.	Sexually maturing males	Aliaga-Guerrero et al. (24)
Pituitary explants and brain slides cultured in the presence of zGnIH-III (0 or 100 nM) and GnRH2 (0 or 100 nM).	*Astyanax altiparanae*	zGnIH-III decreased GnRH2-stimulatory effect on *fshβ and lhβ* levels zGnIH-III alone, or in the presence of GnRH2, stimulated *gnrh2* expression.	Adult males at spawning capable phase.	Branco et al. (78)

*α-gth, α-gonadotropin subunit; BM, body mass; BW, body weight; icv, intracerebroventricular; ip, intraperitoneal; im, intramuscular; gGnIH, goldfish GnIH; grGnIH, orange-spotted grouper GnIH; sbGnIH, sea bass GnIH; ssGnIH, sole senegalensis GnIH; zGnIH, zebrafish GnIH*.

On the other hand, GnIH can stimulate, inhibit, or even have no effect on the synthesis of GnRH variants (Table 1). These discrepancies could be due to differences in sexual stage, route of administration, sampling times and brain regions analyzed. Also, based on studies performed in birds and mammals, it has been proposed that GnIH could indirectly modulate the expression and activity of brain aromatase regulating estradiol levels locally [for review see (79)].

Nowadays, further studies appear necessary to clarify GnIH actions over gonadotropin secretion and/or synthesis in fish. According to anatomical and physiological results presented in this section, it must be emphasized that GnIH interaction with GnRH occurs in fish, and either directly or indirectly GnIH can stimulate or inhibits hypothalamic-pituitary axis depending on the reproductive state of individuals.

## Ontogeny of GnIH System

As Sandvik et al. (84) referred, although RFamide peptides are poorly studied during development, the few reports available in the field show interesting results indicating that many of these peptides have different roles in early stages and in adults. The few studies addressing GnIH ontogeny show that this peptide is not an exception. To date, there are only four studies analyzing the GnIH expression pattern during fish development, showing that this peptide is detected from early developmental stages (33, 50, 51, 53). In zebrafish *gnih* and *gnih-r* transcripts were detected from 1-day post-fertilization (dpf) (prime-5 stage) or blastula stage, respectively; however, in this study, no temporal variations in the expression were evaluated (53). In sea bass, *gnih* and *gnih-r* transcripts were detected from 5 dpf, and although the authors did not quantify the expression in stages prior to hatching, two temporal increases in the *gnih* messengers were observed: one from 5 days post-hatching (dph) to 25 dph, when the larva starts exogenous feeding and the gonad is still undifferentiated, and the other by 150 dph during the onset of gonadal differentiation (50). Studies performed in *C. dimerus* showed that *gnih* was first detected at 1 dph, increased from 12 dph and reached a peak at 20 dph, when the development of gonadal primordia occurred (51). As we previously mentioned, in different species one or more GnIH cell clusters were observed apart from that of NPPv. The spatial-temporal expression pattern of these nuclei could suggest different origins or functions during development. For example, in the Indian major carp, GnIH cells were observed in the NPPv and in the olfactory system (epithelium and bulb) at hatching. This mentioned area showed no GnIH-ir in adults, suggesting a role of these cells during development (33). By contrast, in *C. dimerus* GnIH neurons in the NOR was detected by 3 dph, while NPPv cells by 14 dph (51). The cells in the NOR increase in number from 5 dph, coinciding with the time when larvae start to feed exogenously, and continue to increase in number during the development and differentiation of gonadal primordia. In the same direction, it was observed an increase of NPPv cell number during the development and differentiation of the gonadal primordia. Based on these results, it is suggested that GnIH could be involved in the onset of feeding and gonadal development or sex differentiation in teleosts. This new concept is supported by the variations of *gnih* and *gnih-r* levels in sea bass and *C.dimerus* during these critical periods of early gonadal development (50, 51).

On the other hand, during development GnIH fibers innervate different brain regions (33, 51). Particularly in *C. dimerus*, the presence of GnIH fibers was observed reaching the pituitary from 14 dph to 85 dph, but they clearly diminished from 37 dph on (51). Considering that GnRH1 fibers were detected at 30 dph (85), we could speculate a shift in the neuroendocrine control of pituitary function occurring before gonadal differentiation. The fact that no GnIH fibers innervating the pituitary gland were detected in adults of this species (25), could imply that this neuropeptide would act differently in larvae and adults.

Biotic and abiotic factors, especially temperature and photoperiod, are critical features that could irreversibly affect different biological aspects during development. Even though the neuroendocrine system integrates environmental information, little is known about its development and how it is altered by these factors. For example, low or high incubation temperatures during early developmental stages determine different sex ratio of pejerrey and sea bass larvae (86, 87) indicating that the reproductive axis, at some point, has been altered. To our knowledge, only one study reported the effect of temperature and photoperiod on GnIH system during development (50). In this study, sea bass reared at high temperature, showed a decrease in the expression of *gnih* and *gnih-r*, suggesting that this neuropeptide could be involved in the reported effect of temperature on sex differentiation. Moreover, a seasonal shift in a daily variation of GnIH system related to the reproductive season was demonstrated, indicating the influence of the photoperiod on this system (50). In summary, GnIH in fish development is an almost unexplored area, so more studies are needed in order to further elucidate its role at this particular stage.

## Expanding GnIH Functions Beyond Reproduction

Since the discovery of GnIH, most studies have analyzed the effect of this peptide on the reproductive axis, leaving aside their possible role in the regulation of other functions. In this sense, neuroanatomical localization studies showed in all fish species analyzed that GnIH fibers are broadly distributed along the nervous system, not only in the preoptic-hypothalamic area but also in the retina-optic tract and midbrain, suggesting a potential role of GnIH as neuromodulator or neurotransmitter. In sea bass, GnIH seems to participate in the regulation of fish behavior, as their administration affected the diurnal/nocturnal ratio of locomotors activity during the reproductive cycle (48, 77). It is important to highlight that in this species a cluster of GnIH cells was observed in the midbrain innervating sensory-motor areas (29). On the other hand, there is increasing evidence regarding the effect of GnIH on the synthesis and release of GH (25, 26, 57, 75, 76, 88). Usually, after GnIH administration different responses on the GH synthesis and release were observed depending on the species or the experimental approach. For example, *in vitro* administration of GnIH stimulated GH release in sockeye salmon and *C. dimerus* (25, 26) while in grass puffer, GnIH can increase the abundance of *gh* messengers (88). However, icv administration of GnIH decreased *gh* in sea bass (76), whereas intraperitoneal administration of GnIH did not affect GH release in tilapia (57). However, also concerning to GH regulation, a clear dependence on the reproductive status and on the experimental approach was observed in goldfish (75). In conclusion, these results indicate that GnIH exerts complex effects on basal and GnRH-stimulated somatotrope function in a seasonal-reproductive manner, and thus, this peptide could be involved in the regulation of somatic growth and/or in the interaction between growth and reproduction.

Finally, the ventral telencephalon and the NPPv also exhibit neuropeptide Y (NPY) producing cells in several fish species (89–91). NPY has been implicated in the modulation of gonadotropin release, but also in the regulation of feeding and growth (38, 92). Since GnIH cells are observed in these regions, it is suggested that this neuropeptide could establish a crosstalk among growth, feeding, and reproductive axes. Whether GnIH and NPY are interacting to modulate reproduction, feeding and growth in fish remains to be elucidated.

In conclusion, although the effects of GnIH on reproduction are very clear in birds and mammals, there are still some inconsistencies in fishes that should be addressed soon. Because of our attempt to generalize GnIH function, it is possible that different modes of action or other roles beyond reproduction are leaving aside in fishes. Moreover, as reproduction is a complex event that involves the integration of internal and external cues, it is possible that GnIH acts as a link among them. Interestingly, recent mutation studies for reproductive neuroendocrine factors have shown that, contrary to mammals, kisspeptin, and GnRH null fish can reproduce normally, suggesting a compensatory multifactorial neuroendocrine control of reproduction (93–97). Notably, they found an up-regulation of different neuropeptides involved in the control of reproduction in zebrafish including GnIH (97). According Marvel et al. (97), the up-regulation of GnIH messengers could be related to the action of GnIH as a stimulator of pituitary gonadotropins, as it was already demonstrated in some teleost fish species (25, 74). Further studies are still required in order to clarify the role of GnIH in teleosts including its involvement in development, a key stage that strongly impacts on adult biology.

## Author Contributions

MD, JM-C, JP-S, GS, KT, and PV contributed equally to the manuscript and approved it for publication.

### Conflict of Interest Statement

The authors declare that the research was conducted in the absence of any commercial or financial relationships that could be construed as a potential conflict of interest.
